# A Comparison of the Performance of the I-gel™ vs. the LMA-S™during Anesthesia: A Meta-Analysis of Randomized Controlled Trials

**DOI:** 10.1371/journal.pone.0071910

**Published:** 2013-08-12

**Authors:** Xiaoguang Chen, Jinghua Jiao, Xuefeng Cong, Lei Liu, Xiaomei Wu

**Affiliations:** 1 Department of Anesthesiology, The First Hospital of China Medical University, Shenyang, China; 2 Department of Anesthesiology, Fengtian Hospital of Shengyang Medical College, Shenyang, China; 3 Department of Neurosurgery, The First Hospital of Shenyang, Shenyang, China; 4 Department of Ophthalmology, The First Hospital of China Medical University, Shenyang, China; 5 Department of Clinical Epidemiology and Evidence Medicine, The First Hospital of China Medical University, Shenyang, China; University of Colorado, United States of America

## Abstract

**Background and Objective:**

Conflicting results were found between the I-gel™ and the LMA-Supreme™ during anesthesia, so we conducted a meta-analysis of randomized controlled trials (RCTs) to compare the effectiveness and safety of the I-gel™ vs. the LMA-Supreme™during anesthesia.

**Methods:**

A comprehensive search was conducted using Pubmed, EMbase, ISI Web of Knowledge, the Cochrane Library, China Journal Full-text Database, Chinese Biomedical Database, Chinese Scientific Journals Full-text Database, CMA Digital Periodicals, and Google scholar to find RCTs that compare the LMA-S™ with the i-gel™during anesthesia. Two reviewers independently selected trials, extracted data, and assessed the methodological qualities and evidence levels. Data were analyzed by RevMan 5.0 and comprehensive meta-analysis software.

**Results:**

Ten RCTs were included. There were no significant differences in oropharyngeal leak pressures (mean difference [MD] 0.72, 95% confidence interval [CI] –1.10 2.53), device placement time (MD –1.3, 95%CI –4.07 1.44), first attempt insertion success (risk ratio [RR] 1.01, 95% CI 0.9 1.14), grade 3 and 4 fiberoptic view (RR 0.89, 95%CI 0.65 1.21), and blood on removal (RR 0.62, 95%CI 0.32 1.22) between the i-gel™ and the LMA-Supreme™, respectively. However, the LMA-Supreme™was associated with easier gastric tube insertion (RR 1.17, 95%CI 1.07 1.29), and more sore throat (RR 2.56, 95%CI 1.60 4.12) than the i-gel™ group.

**Conclusions:**

The LMA-Supreme™ and i-gel™ were similarly successful and rapidly inserted. However, the LMA-Supreme™ was shown to be easier for gastric tube insertion and associated with more sore throat compared with the i-gel™.

## Introduction

Since the introduction of the classic laryngeal mask airway, the field of supraglottic airway devices (SGA) has experienced a remarkable evolution and SGA are now routinely used in clinical anaesthesia [1]. The i-gel™ (Intersurgical Ltd, Wokingham, Berkshire, United Kingdom) and the Laryngeal Mask Supreme™ (LMA-S™; Laryngeal Mask Company, Henley-on-Thames, United Kingdom) are two single-use supraglottic airway devices. The i-gel™comprises a soft, gel-like, non-inflatable cuff made of thermoplastic elastomer, a widened, flattened stem with a rigid bite-block that acts as a buccal stabilizer to reduce axial rotation and malpositioning, and an oesophageal vent through which a gastric tube can be passed [2]–[4]. Whereas the LMA Supreme™ is an inflatable device with an oesophageal drainage tube for suctioning gastric contents, with similar characteristics to the i-gel™: presence of a drain tube to separate the gastrointestinal tract from the respiratory tract and built-in bite block [4]–[6]. It differs from the i-gel™ in the following aspects: it is constructed of medical grade silicone, and has an inflatable cuff, a reinforced tip, and an elliptical, anatomically shaped, semi-rigid airway tube [4]. Their potential advantages include easier insertion, better airway protection, more effective ventilation, and better fiberoptic view of the glottis [3], [6], [7].

There has been a lot of interest in these two devices due to their acclaimed advantages, and there have been a number of studies in response to concerns regarding their effectiveness and safety [1], [5]–[9]. However, there have been conflicting results concerning oropharyngeal leak pressure, ease of insertion, and adverse effects of these two devices during anesthesia. Regarding to first attempt insertion success and mean oropharyngeal leak pressure, they were significantly higher in the LMA Supreme™ than the i-gel™ [10], but not in other studies [6]–[8]. To our knowledge, there has been no previous meta-analysis comparing these two devices. Thus, we conducted a meta-analysis comparing these two devices with a detailed evaluation of their effectiveness and safety during anesthesia.

## Methods

We did this meta-analysis of available randomized controlled trials (RCTs) in accordance with the PRISMA guidelines [11].

### Search Strategy

Systematic literature searches were conducted in PubMed, the Cochrane library, EMBASE, ISI Web of Knowledge, China Journal Full-text Database, Chinese Biomedical Database, Chinese Scientific Journals Full-text Database, CMA Digital Periodicals, and Google scholar. Search terms included laryngeal mask, LMA-S, LMA Supreme, i-gel. All searches were conducted in May 2012, and updated in December 2012. Reference lists of relevant reviews and eligible articles were hand-searched. A search of the ClinicalTrials.gov website was also conducted to identify RCTs which were completed but not yet published. Requests for original papers that were not published were made by contacting authors or principal investigators. All searches were conducted independently by two reviewers (JH Jiao and XG Chen); differences were resolved by discussion.

### Inclusion and Exclusion Criteria

Reports potentially eligible for this meta-analysis had to meet the following criteria: they had to be RCTs, written in English or Chinese, and the studies needed to provide sufficient information to pool the effectiveness and safety of the LMA-Supreme™ and i-gel™. The outcomes that we evaluated included oropharyngeal leak pressure, grade 3 and 4 fiberoptic view, device insertion time, first attempt insertion success, ease of gastric tube insertion, blood on removal, and sore throat. Articles were excluded if they did not satisfy one or more inclusion criteria.

### Study selection and data Extraction

Two reviewers (XF Cong and L Liu) independently assessed potential citations for inclusion; disagreements were resolved with a third reviewer (XG Chen). Data was extracted from each article using a standardized form by two independent reviewers (XM Wu and L Liu) to abstract the following information: country, patient characteristics (age, sex, etc), and treatment protocols (details of intervention & comparison, sample size, etc), and outcomes. Outcomes were extracted preferentially by intention to treat method. Any disagreements were resolved with a third reviewer (XG Chen).

### Quality and evidence level assessment

Methodological quality was assessed by the Cochrane handbook 5.0 recommended standard [12]: randomization, blinding, concealed allocation, selective reporting, incomplete outcome data, and other biases. For evaluating the evidence levels of the outcomes, we used the Grading of Recommendations Assessment, Development and Evaluation (GRADE) approach, which specifies four levels: high, moderate, low, and very low [13]. Two reviewers (XF Cong and JH Jiao) independently assessed quality for each RCT and evidence level for each outcome; disagreements were resolved with a third reviewer (XG Chen).

### Data Analysis

RevMan 5.0 software was used to conduct the meta-analysis. We computed the relative risk (RR) with corresponding 95% confidence interval (CI) for dichotomous outcome data, and mean difference (MD) with its 95%CI for continuous variable. The percentage of variability across trials attributable to heterogeneity beyond chance was estimated with the I^2^ statistic, which was deemed significant when p was less than 0.05 or I-square was more than 50% [14]. Data was pooled using both the fixed-effect model and the random-effect model.

Important variables such as country, surgery or patients’ condition, sample size, pre-anesthetic medication, and general anesthesia methods could affect the pooled results. So we conducted mixed meta-regression regression (unrestricted maximum likelihood) to investigate their influences on the primary outcomes. The meta-regression was conducted using comprehensive meta-analysis software 2.0.

In order to find the influence of methodological quality on the outcomes, we also pooled results using quality effect model by metaXL software. And the Q index (Qi) was calculated according to suggested quality scoring system for both experimental and prospective observational studies [15]. Publication bias was assessed by visually inspecting a funnel plot. The small-study effect in terms of publication bias was also estimated using Egger's linear regression test [16].

## Results

### Search results

**Figure 1:**
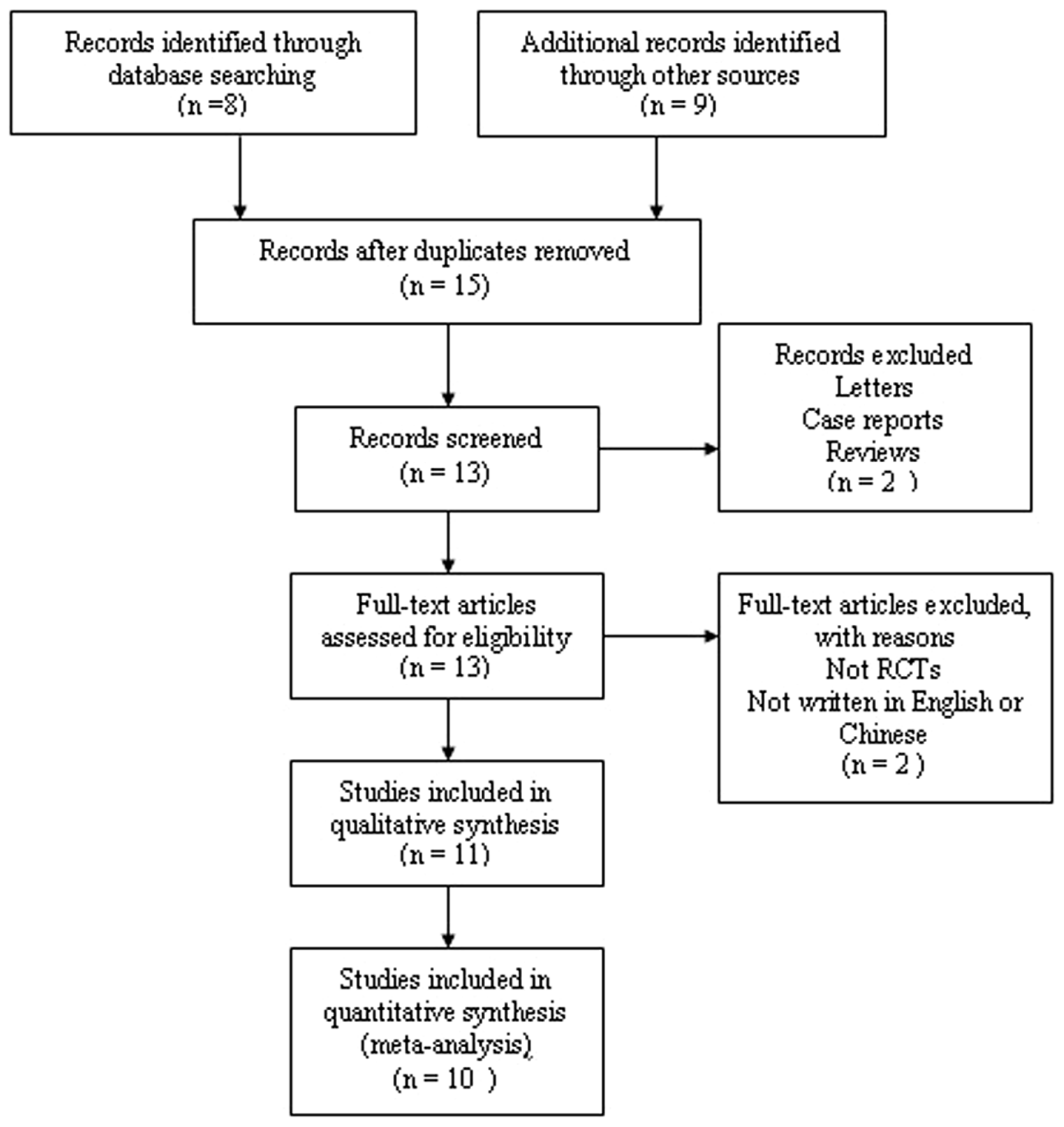
Flow chart demonstrating the process for inclusion in the meta-analysis.

A total of 168 records were identified after comprehensive searches. No records were derived from ClinicalTrials.gov and contacting authors. Based on screening titles and abstracts, we excluded duplicates (n = 42), studies that did not include both LMA-S and i-gel (n  = 54), and studies that were not RCTs (n  = 50). Then based on reading full-texts we excluded studies those did not included both LMA-S and i-gel (n  = 7) and studies that were not RCTs (n  = 5). Finally, ten studies [1], [4], [8]–[10], [17]–[21] were included. The progress for study selection is shown in Figure 1.

### Characteristics of included trials

Ten studies were from China (n = 3), Germany (n = 2), Italy (n = 1), Singapore (n = 1), Switzerland (n = 1), The Netherlands (n = 1), and Malaysia (n = 1).

The surgeries the patients underwent were elective surgery ( 2 studies), laparoscopic gynecological surgery (2 studies ), elective peripheral or superficial surgery (1 study), spontaneously breathing anesthetized patients (1 study), laparoscopic gynecological and laparoscopic cholecystectomy (1 study), paralyzed anesthetized patient (1 study), laparoscopic cholecystectomy (1 study), and breast surgery (1 study). The sample size ranged from 30 to 150 with a total number of 860. And other information was presented in Table 1.

**Table 1:** Summary of included studies.

Study	Country	N	Age (year)	Weight (Kg)	Surgery or patients’ condition	ASA grading	Pre-anesthetic medication	General anesthesia	Primary measurement	how OP leak pressures were obtained	Definition for the inflation protocols for the LMA-Supreme
Cao 2012 [19]	China	50	20–60	50–80	Laparoscopic gynecological and Cholecystectomy	I–II	atropine+ midazolam	fentanyl+propofol	oropharyngeal leak pressure	using the technique of static equilibrium	inflated by volume
Chew 2010 [8]	Malaysia	90	16–55	N/A	Spontaneously breathing anesthetized patients	I–II	N/A	fentanyl+propofol+sevoflurane	oropharyngeal leak pressure	using the technique of static equilibrium	inflated by volume
Eschertzhuber 2012 [18]	Germany	30	20–70	N/A	Paralyzed anesthetized patient	I–II	midazolam	fentanyl+propofol+rocuronium	oropharyngeal leak pressure	using the technique of static equilibrium	inflated by volume
Li 2011 [20]	China	120	30–60	45–90	Laparoscopic Cholecystectomy	I–II	midazolam + atropine	fentanyl+vecuronim	oropharyngeal leak pressure	using the technique of static equilibrium	inflated by volume
Ragazzi 2012 [10]	Italy	80	18+	N/A	Breast surgery	I–III	without	remifentanil+propofol	insertion success rate	unclear	unclear
Russo 2012 [1]	Germany	120	18+	N/A	Elective surgery	I–II	midazolam	sufentanil+propofol	oropharyngeal leak pressure	determined as a function of cuff pressure for the supraglottic airway devices with inflatable cuffs	inflated by volume
Teoh 2010 [4]	Singapore	100	20–80	30+	Laparoscopic gynecological	I–II	without	fentanyl+propofol+atracurium	oropharyngeal leak pressure	using the technique of static equilibrium	unclear
Theiler 2009 [17]	Switzerland	60	18–80	50+	Elective surgery	I–III	midazolam	fentanyl+propofol+remifentanil	Overall success rate	unclear	unclear
Van Zundert 2012 [9]	The Netherlands	150	18–80	50–120	elective peripheral or superficial surgery	I–II	N/A	fentanyl+propofol+sufentanil	ease of insertion	using the technique of static equilibrium	inflated by volume
Yu 2011 [21]	China	60	21–64	45–90	Laparoscopic gynecological	I–III	N/A	propofol+remifentanil+rocuronium	oropharyngeal leak pressure	using the technique of static equilibrium	inflated by volume

### Quality and evidence level assessment

Ten studies mentioned randomization, but only seven studies reported the details of randomization, and four studies reported the details of concealed allocation. Four studies mentioned blinding, and only three mentioned who were blinded to. And other information was presented in Table 2.

**Table 2:** Quality assessment of included studies.

	randomization	concealed allocation	blinding	incomplete outcome data	selective reporting	other biases	Qi
Cao 2012	mentioned	unclear	unclear	unclear	unclear	unclear	0.75
Chew 2010	mentioned	yes, sealed opaque envelopes	no	unclear	unclear	yes, conflict of interest	0.83
Eschertzhuber 2012	mentioned	unclear	yes	unclear	unclear	unclear	0.92
Li 2011	yes, random number	unclear	unclear	unclear	unclear	unclear	0.75
Ragazzi 2012	yes, random number	unclear	unclear	unclear	unclear	unclear	0.83
Russo 2012	yes, random number	yes, sealed opaque envelopes	unclear	unclear	unclear	no	0.83
Teoh 2010	yes, random number	yes, sealed opaque envelopes	blinded to patients	unclear	unclear	yes, conflict of interest	0.96
Theiler 2009	yes, random number	unclear	blinded to operators	unclear	unclear	yes, conflict of interest	0.88
Van Zundert 2012	yes, random number	yes, sealed opaque envelopes	blinded to observer	unclear	unclear	unclear	0.96
Yu 2011	yes, random number	unclear	unclear	unclear	unclear	unclear	0.83

For the seven outcomes we evaluated, the evidence levels for two outcomes (grade 3 and 4 fiberoptic view and device insertion time) were very low, and the evidence levels for the rest were low, due to high risk of bias, high heterogeneity, or small sample size (Table 3).

**Table 3:** Meta-analysis results for each outcome.

	Fixed-model	Random-model	Quality-effect model	Heterogeneity	Evidence level
Oropharyngeal leak pressure	MD 0.46, 95%CI (–0.32 1.23)	MD 0.72, 95%CI (–1.10 2.53)	MD 0.61, 95%CI (–0.25 1.46)	I^2^ = 79%, P < 0.00001	Low
Grade 3 and 4 fiberoptic view	RR 0.87, 95%CI (0.76 0.99)	RR 0.88, 95%CI (0.63 1.22)	RR 0.90, 95%CI (0.68 1.19)	I^2^ = 84%, P < 0.0001	Very low
Device insertion time	MD –0.05, 95%CI (–1.16 1.06)	MD –1.30, 95%CI (–4.02 1.44)	MD –0.51, 95%CI (–1.76 0.75)	I^2^ = 75%, P = 0.003	Very low
First attempt insertion success	RR 1.08, 95%CI (1.00 1.16)	RR 1.05, 95%CI (0.97 1.13)	RR 1.04, 95%CI (0.97 1.12)	I^2^ = 69%, P = 0.006	Low
Ease of gastric tube insertion	RR 1.17, 95%CI (1.07 1.29)	RR 1.18, 95%CI (1.06 1.31)	RR 1.18, 95%CI (1.08 1.32)	I^2^ = 45%, P = 0.16	Low
Sore throat	RR 2.55, 95%CI (1.59 4.09)	RR 2.45, 95%CI (1.50 3.99)	RR 2.36, 95%CI (1.43 3.88)	I^2^ = 0%, P = 0.53	Low
Blood on removal	RR 0.62, 95%CI (0.32 1.22)	RR 0.61, 95%CI (0.27 1.41)	RR 0.58 95%CI (0.27 1.22)	I^2^ = 16%, P = 0.31	Low

### Meta analysis results

**Figure 2:**
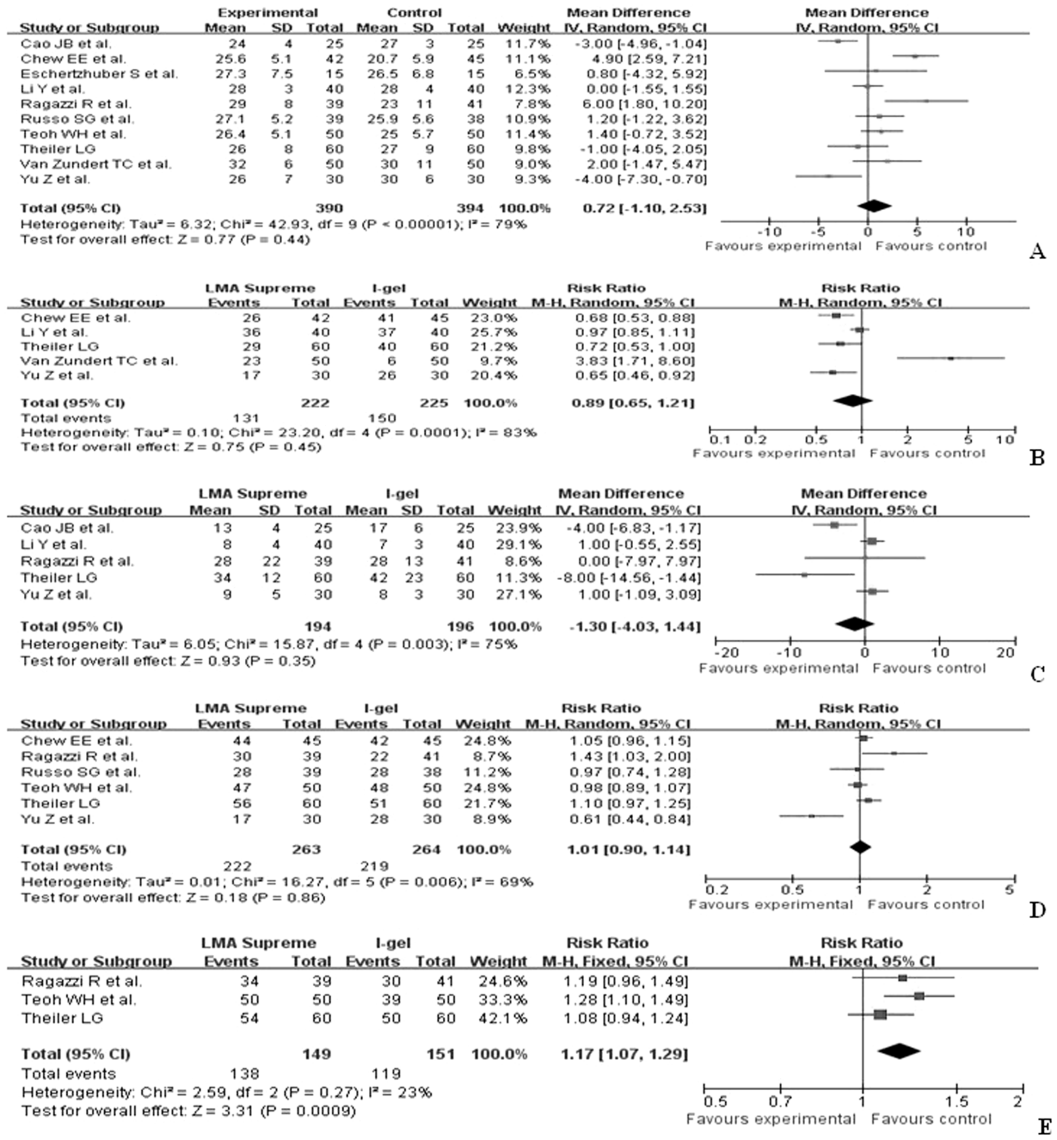
Forest plot displaying the pooled summary performance of the LMA-Supreme™ versus the i-gel™: A) oropharyngeal leak pressure, B) grade 3 and 4 fiberoptic view, C) device insertion time, D) first attempt insertion success, E) ease of gastric tube insertion. SD = standard deviation; IV = weighted mean difference; CI = confidence interval; df = degrees of freedom; Chi^2^ = chi-square statistic; p = p value; I^2^ = I-square heterogeneity statistic; Z = Z statistic; RR = risk ratio; WMD =  weight mean difference.

The pooled results revealed that there were similar oropharyngeal leak pressures during anesthesia between the LMA-Supreme™and the i-gel™ (WMD 0.72, 95%CI –1.10 2.53). There were no significant differences in device placement time (WMD –1.3, 95%CI –4.07 1.44), first attempt insertion success (RR 1.01, 95%CI 0.9 1.14), and grade 3 and 4 fiberoptic view (RR 0.89, 95%CI 0.65–1.21) between the LMA-Supreme™ group and the i-gel™ group. However, gastric tube insertion was significantly easier (RR 1.17, 95%CI 1.07 1.29) in the LMA-Supreme™than that in the i-gel™group during anesthesia (Figure 2).

**Figure 3:**
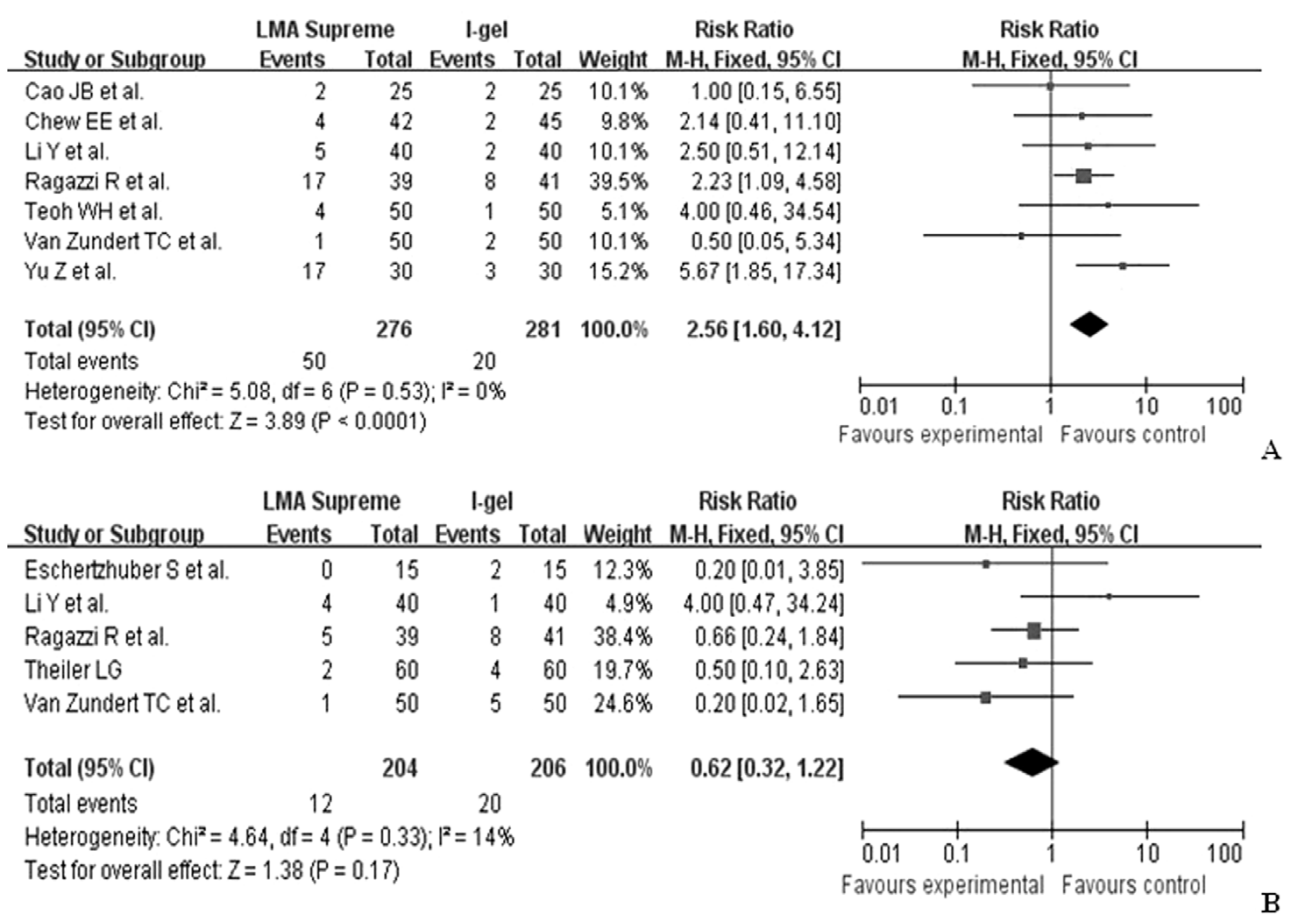
Forest plot displaying the pooled summary of adverse effects of the LMA-Supreme™ versus the i-gel™: A) sore throat, B) blood on removal. SD = standard deviation; IV = weighted mean difference; CI = confidence interval; df = degrees of freedom; Chi^2^ = chi-square statistic; *p* = *p* value; I^2^ = I-square heterogeneity statistic; Z = Z statistic; RR = risk ratio; WMD =  weight mean difference.

There was no significant difference in the presence of blood on device removal between the LMA-Supreme™ and the the i-gel™ (RR 0.62, 95%CI 0.32 1.22), but sore throat was more common in the LMA-Supreme™ group than that in the i-gel™ group (RR 2.56, 95%CI 1.60 4.12) (Figure 3).

Both fixed- and random- effects models were used to pool the results, and the results for seven outcomes were consistent (Table 3). We also took quality into account using quality effect models. And the results produced by quality effect models were also in accordance with the results by both fixed- and random- effects models (Table 3).

### Meta-regression results

The results of meta-regression showed that coefficients for country, surgery or patients’ condition, sample size, pre-anesthetic medication and general anesthesia methods were not statistically significant. (Table 4)

**Table 4:** Meta-regression results of variables for oropharyngeal leak pressure.

	Coefficient	95%CI		P	Residual Q
Country	0.04	–0.03	0.10	0.26	36.99
Surgery or patients’ condition	0.04	–0.11	0.19	0.62	10.18
Sample size	0.01	–0.00	0.01	0.17	10.30
Pre-anesthetic medication	0.08	–0.09	0.24	0.35	10.02
General anesthesia	–0.01	–0.15	0.12	0.86	10.34

### Publication bias

**Figure 4:**
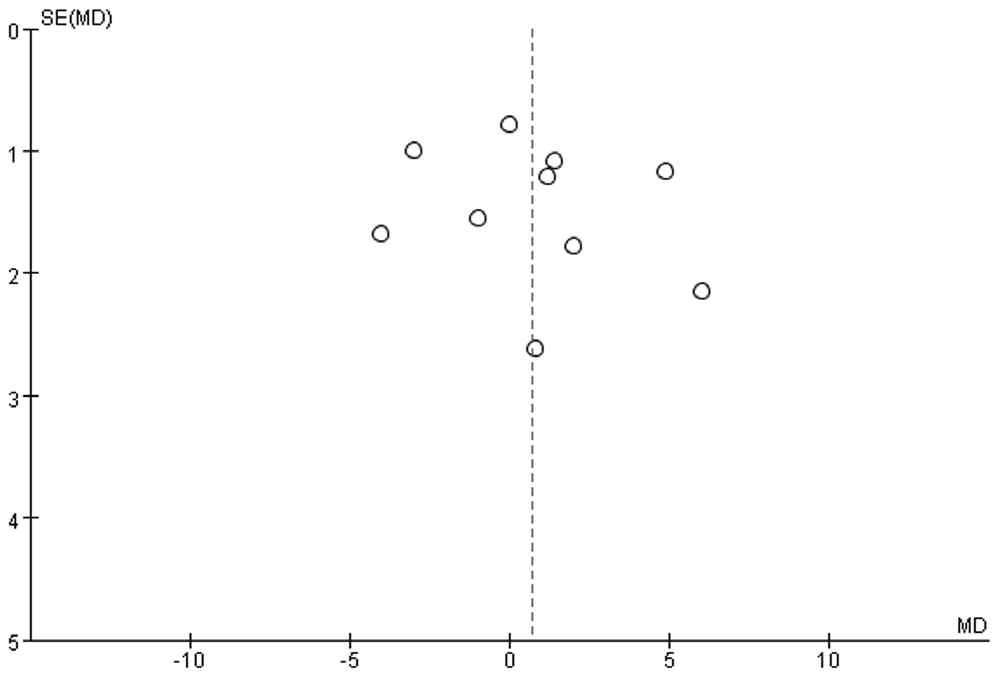
Funnel plot of randomized controlled trials.

There was no significant publication bias based on funnel plot (Figure 4). Egger's test indicated that there was not a possibility of publication bias for oropharyngeal leak pressures (intercept –3.45, 95%CI –12.07 5.16, p = 0.38).

## Discussion

### Summary of finding

This meta-analysis summarized evidence in published RCTs concerning the effectiveness and safety of the LMA-Supreme™ vs. the i-gel™ during anesthesia. The LMA-Supreme™ was similar to the i-gel™in oropharyngeal leak pressure, first attempt insertion success, device insertion time, grade 3 and 4 fiberoptic view and blood on removal. However, LMA-Supreme™was associated with easier gastric tube insertion and more sore throat than the i-gel™. Country, surgery or patients’ condition, sample size, pre-anesthetic medication, and general anesthesia did not affect the pooled results of the oropharyngeal leak pressure between the LMA-Supreme™and the i-gel™. However, the methodological quality of included studies was of high risk and the evidence levels for outcomes were low or very low.

Six RCTs in this analysis [1], [4], [8], [10], [18], [22] demonstrated positive trends which is consistent with the pooled result of oropharyngeal leak pressures in our meta-analysis. The oropharyngeal leak pressures were similar for both devices based on these six RCTs and the results of this meta-analysis. However, other studies [19], [21] have suggested that the i-gel™ provided a higher oropharyngeal leak pressure than the LMA-Supreme™. On the other hand, Chew et al. [8] and Ragazzi et al. [10] demonstrated that oropharyngeal leak pressures were significantly greater with the LMA-Supreme™ than those with the i-gel™.

Our meta-analysis showed no significant difference on number of grade 3 and 4 fiberoptic view between the i-gel™ and the LMA-Supreme™. This suggests that these two devices might function similarly as a conduit in difficult airway management and failed intubation. The LMA-Supreme™ has an anatomically shaped airway tube, a drain tube (DT), a modified inflatable cuff, an integral bite block, and a fixation tab [23]. The DT provides access to gastric contents [24] and is helpful for gastric tube insertion, but we found that there were no significant differences in first attempt insertion success rates and speed of insertion, which means that the two devices a appeared equally easy to insert in patients under anesthesia [22], [25].

Regarding to safety, there was not a significant difference in blood on removal between the two devices. However, sore throat was more in LMA-Supreme™than that in the i-gel ™. The I-gel ™ has non-inflatable cuff seals that fit anatomically against perilaryngeal structures [26], but the LMA-Supreme™ has been designed with an inflatable cuff and a reinforced tip. Yu et al. [9] and Ragazzi et al. [4] reported that sore throat was more common in the LMA-Supreme™ group than that in the i-gel™ group, which is consistent with our meta-analysis result. This complication may be related to the inflatable cuff of the LMA-Supreme™ compressing microvascular structures and terminal nerve endings in these tissues [2].

In conclusion, both the LMA-Supreme™ and the i-gel™ were similarly successful and rapidly inserted. However, gastric tube insertion was much easier with the LMA-Supreme™ than that with the i-gel™. Sore throat was more common with the LMA-Supreme™ than that with the i-gel™.

### Strength and limitation

To the best of our knowledge, this is the first meta-analysis specifically evaluating the effectiveness and safety of the LMA-Supreme™ and the i-gel™ during anesthesia. However, there were several limitations of this study: (1) Although we conducted meta-regression of different surgeries, countries, sample size, pre-anesthetic medication, and general anesthesia methods, country, surgery or patients’ condition, sample size, pre-anesthetic medication and general anesthesia methods did not affect the results of oropharyngeal leak pressure. Many factors including patient characteristics, depths of anaesthesia, successes of insertion, paralyzed statuses and the settings could affect the anesthetic effects. However, we did not conduct meta-regression of these factors due to limited data.

(2) Our results revealed that there might be heterogeneity. These factors we mentioned above probably contributed to the heterogeneity between studies. It was difficult to adequately scrutinize the heterogeneity of outcomes between studies, as the nuanced differences between the studies were not defined or appreciated. The heterogeneity across studies was clinical heterogeneity and might be the biggest issue in this meta-analysis. The end points are faulted and have inherent error that cannot be quantified, which would lower the reliability of the data among studies. In this condition, it is hard to judge that the effects observed is the true effect or not. More often, the effects could be affected by the clinical heterogeneity among studies and the influence of clinical heterogeneity for end points should be investigated. However, we could not investigate their influence by conducting subgroup analysis or meta-regression analysis due to limited data.

(3) Although we have tried to access to un-published results, we did not include any unpublished studies. (4) For oropharyngeal leak pressure using supraglottic airway devices, it depends significantly on the body habitus, the experience of the operator and assistant, and the depth of anaesthesia. But none of our included studies reported the influence of body habitus and the experience of the operator and assistant on oropharyngeal leak pressure. Failure to include such clinically important measures limits the usefulness of this entire manuscript. So in the future, better RCTs should include such data. (5) Due to small sample size of included studies, high heterogeneity across the included studies and high risk of bias of included studies, the evidence level for the evidence was low. (6) Although the personal preference might influence the choice of LMA, there is no data to quantify how much personal preference influenced the choice of LMA.

### Implication for research and practice

The sample sizes of included studies were not large enough, ranging from 30 to 150. The number of participants in clinical research should always be large enough to provide a sufficiently precise answer to the research question posed, so in the future the sample size for the RCTs comparing LMA-Supreme™ with i-gel™should be large enough to detect the small differences.

The methodological quality for all included studies was not high, as some important methodological items were not well conducted or reported, such as concealed allocation. All the studies mentioned randomization, but not all studies mentioned the details of randomization methods and concealed allocation. So in the future, important methodological items such as randomization, concealed allocation and blinding should be well conducted and reported.

At the same time, none of included studies mentioned the influences of patient characteristics, depths of anaesthesia, successes of insertion, paralyzed statuses and the settings. Only one study reported the effectiveness of LMA-Supreme™ vs. i-gel™in paralyzed patients, and its result was consistent with the pooled results, so we thought paralyzed status did not affect the results. Whether these factors affect the the true effects of LMA should be well discussed in the future RCTs. Meanwhile, studies that quantify how much personal preference influenced the choice of LMA should also be conducted.

For practice, our meta-analysis showed that the LMA-Supreme™ was similar to the i-gel™in oropharyngeal leak pressure, first attempt insertion success, device insertion time, grade 3 and 4 fiberoptic view and blood on removal with easier gastric tube insertion and more sore throat. So in the future, anesthetists should choose different devices according to the real situations.

## Conclusion

This meta-analysis summarized evidence in published RCTs concerning the effectiveness and safety of the LMA-Supreme™ vs. the i-gel™ during anesthesia. The LMA-Supreme™ was similar to the i-gel™in oropharyngeal leak pressure, first attempt insertion success, device insertion time, grade 3 and 4 fiberoptic view and blood on removal. However, gastric tube insertion was easier and sore throat was more in LMA-Supreme™than those in the i-gel™. Even with the limitations, we feel the conclusions of this meta-analysis are clinically useful for the consideration of anesthetists.

## Supporting Information

Table S1 **PRISMA 2009 Flow Diagram.**
(DOC)

Table S2 **PRISMA 2009 Checklist.**
(DOC)
