# Supplementary material for: A Comparison of the Performance of the I-gel™ vs. the LMA-S™during Anesthesia: A Meta-Analysis of Randomized Controlled Trials
Source: PLoS One. 2013 Aug 12;8(8):e71910. doi: 10.1371/journal.pone.0071910 (PMC3741209; doi:10.1371/journal.pone.0071910)
Supplement: Table S1 — PRISMA 2009 Flow Diagram. (DOC) [file pone.0071910.s001.doc]

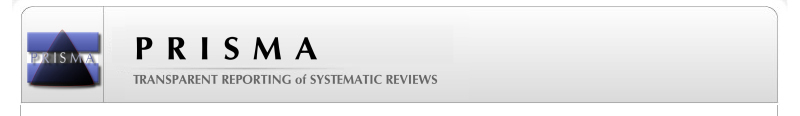
**PRISMA 2009 Flow Diagram**

**Screening**

**Included**

**Eligibility**

**Identification**

Records identified through database searching
(n =8)

Additional records identified through other sources
(n = 9)

Records after duplicates removed
(n = 15)

Records screened
(n = 13)

Records excluded

Letters

Case reports

Reviews
(n = 2 )

Full-text articles assessed for eligibility
(n = 13)

Full-text articles excluded, with reasons

Not RCTs

Not written in English or Chinese
(n = 2 )

Studies included in qualitative synthesis
(n = 11)

Studies included in quantitative synthesis (meta-analysis)
(n = 10 )
